# Mesenchymal Stem Cells Isolated from Adipose and Other Tissues: Basic Biological Properties and Clinical Applications

**DOI:** 10.1155/2012/461718

**Published:** 2012-05-14

**Authors:** Hakan Orbay, Morikuni Tobita, Hiroshi Mizuno

**Affiliations:** ^1^Department of Plastic and Reconstructive Surgery, Nippon Medical School, Tokyo 113-0022, Japan; ^2^Department of Dentistry and Oral Surgery, Self Defense Force Hospital, Yokosuka 237-0071, Japan; ^3^Department of Plastic and Reconstructive Surgery, Juntendo University School of Medicine, Tokyo 1138421, Japan

## Abstract

Mesenchymal stem cells (MSCs) are adult stem cells that were initially isolated from bone marrow. However, subsequent research has shown that other adult tissues also contain MSCs. MSCs originate from mesenchyme, which is embryonic tissue derived from the mesoderm. These cells actively proliferate, giving rise to new cells in some tissues, but remain quiescent in others. MSCs are capable of differentiating into multiple cell types including adipocytes, chondrocytes, osteocytes, and cardiomyocytes. Isolation and induction of these cells could provide a new therapeutic tool for replacing damaged or lost adult tissues. However, the biological properties and use of stem cells in a clinical setting must be well established before significant clinical benefits are obtained. This paper summarizes data on the biological properties of MSCs and discusses current and potential clinical applications.

## 1. Introduction

A stem cell is an undifferentiated cell with the capacity for multilineage differentiation and self-renewal without senescence. Totipotent stem cells (zygotes) can give rise to a full viable organism and pluripotent stem cells (embryonic stem (ES) cells) can differentiate into any cell type within in the human body. By contrast, trophoblasts are multipotent stem cells that can differentiate into some (e.g., mesenchymal stem cells (MSCs), hematopoietic stem cells (HSCs)), but not all, cell types.

**Figure 1:**
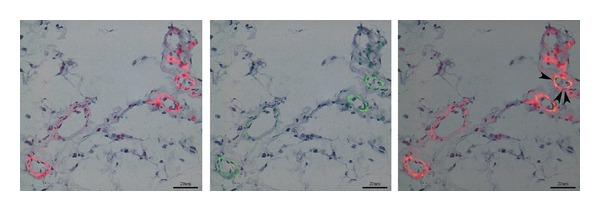
Double immunofluorescence staining of microvessels in a mouse inguinal fat pad (paraffin embedded). CD34-positive (*left; secondary antibody Texas red*) and *α*-smooth muscle actin-positive (*α*-SMA, *middle; secondary antibody FITC*) staining is shown. The cells surrounding the microvessels are positive for both CD34 and *α*-SMA (*right panel: *marked with black arrow heads), suggesting a possible relationship between pericytes and MSCs. Nuclei were counterstained with hematoxylin. Scale bars, 20 *μ*m.

Adult tissues have specific stem cell niches, which supply replacement cells during normal cell turnover and tissue regeneration following injury [1–3]. The epidermis, hair, HSCs, and the gastrointestinal tract all present good examples of tissues with niches that contribute stem cells during normal cellular turnover [3]. The exact locations of these stem cell niches are poorly understood, but there is growing evidence suggesting a close relationship with pericytes [1, 4, 5] (Figure 1). MSCs have been isolated from adipose tissue [6], tendon [7], periodontal ligament [8], synovial membranes [9], trabecular bone [10], bone marrow [11], embryonic tissues [12], the nervous system [13], skin [14], periosteum [9], and muscle [15]. These adult stem cells were once thought to be committed cell lines that could give rise to only one type of cell, but are now known to have a much greater level of plasticity [16, 17]. Despite the vast variety of source tissues, MSCs show some common characteristics that support the hypothesis of a common origin [1, 18]. These characteristics are: fibroblast like shape in culture, multipotent differentiation, extensive proliferation capacity, and a common surface marker profile (e.g., CD34^−^, CD45^−^(HSC markers), CD31^−^ (endothelial cell marker), CD44^+^, CD90^+^, and CD105^+^ (Table 1)). However, there is no surface marker that uniquely defines MSCs.

The same general approaches are used to isolate all kinds of MSCs, including the use of Dulbecco's Modified Eagle Medium (DMEM) to dissolve collagenase, digestion times limited to a maximum of 1 hour at 37°C, isolation of stem cells as soon as possible following euthanasia, and the use of culture medium at temperatures not lower than room temperature [1].

Numerous studies have been conducted by different researchers from different scientific disciplines using stem cells. However, the results are somewhat inconsistent, which has led to a number of controversies in the literature. To review all these controversies along with the underlying data would be an overwhelming task; therefore, the aim of this paper is to briefly describe the biological properties of the main types of MSCs and to discuss their potential clinical applications.

## 2. Adipose-Derived Stem Cells (ASCs)

ASCs were first isolated by Zuk et al. [19]. ASCs can differentiate into ectodermal and endodermal lineages, as well as the mesodermal lineage [20]. ASCs can be obtained from either liposuction aspirates or excised fat. Small amounts of adipose tissue (100 to 200 mL) can be obtained under local anesthesia. One gram of adipose tissue yields approximately 5,000 stem cells, whereas the yield from BM-derived MSCs is 100 to 1,000 cells/mL of marrow [21]. On average, the yield of ASCs from processed lipoaspirate comprises approximately 2% of nucleated cells [21]. In their original study, Zuk et al. noted that ASCs express CD13, CD29, CD44, CD71, CD90, CD105/SH2, SH3, and STRO-1. In contrast, no expression of the hematopoietic lineage markers CD14, CD16, CD31, CD34, CD45, CD 56, CD 61, CD 62E, CD 104, and CD106 was observed [20]. Although ASCs were only identified relatively recently, their ease of harvest and abundance place them in a unique position relative to other MSCs.

## 3. Bone Marrow-Derived-Stem Cells (BM-MSCs)

BM-MSCs are a primitive population of CD34^−^, CD45^−^, CD44^+^, CD105^+^, CD166^+^, CD28^+^, CD33^+^, CD13^+^ and HLA class I^+^ cells [22]. The existence of precursor stromal cells in bone marrow has long been known and these cells were first named Westen-Bainton cells [23]. It was Friedenstein et al., who plated these cells and obtained colony forming units in vitro for the first time [24]. Studies by Castro-Malaspina et al. [25], Fei et al. [26], and Song et al. [27] supplied a better understanding of biological properties of bone marrow stromal cells; such as their fibroblast-like morphology, and the lack of the basic characteristics of endothelial cells and macrophages. Subsequent studies by Chailakhyan and Lalykina [28], Ashton et al. [29], Patt et al. [30], Owen [31], Bennett et al. [32], and revealed the in vitro multipotent differentiation capacity of bone marrow stromal cells. Two milestone studies documenting the multipotential differentiation of BM-MSCs were published by Caplan [33] and Pittenger et al. [34]. Currently, BM-MSCs are known to differentiate into osteogenic, adipogenic, chondrogenic and neural lineages [22, 35]. MSCs in the BM are thought to generate and maintain the proper microenvironment for HSCs by secreting cytokines and growth factors [22, 35, 36]. The estimated frequency of BM-MSCs is 1 in 3.4 × 104 cells, the lowest among the known sources of MSCs [22]. Yoshimura et al. [37] showed that rat BM-MSCs were the least potent stem cell in terms of colony number per nucleated cell, colony number per adherent cell, and cell number per colony. Gronthos et al. [38] suggested two possible origins for BM-MSCs: vascular smooth muscle cells or pericytes (since BM-MSCs express *α*-SMA and respond to PDGF) (Figure 1) or endosteal cells.

The method used to isolate BM-MSCs is different from that used for other MSCs. This is because little extracellular matrix is present in BM; therefore, instead of collagenase digestion, gentle mechanical disruption by repeated pipetting is used to create a suspension of stromal and hematopoietic cells. Upon plating, BM-MSCs rapidly adhere to culture dishes, whereas nonadherent hematopoietic cells are washed away by medium changes [5]. The resultant BM-MSC population is highly heterogeneous and isolating pure stem cells from this primary isolate is difficult due to the lack of unique cell surface markers [5].

## 4. Periodontal Ligament-Derived Stem Cells (PDL-SCs)

The periodontium comprises the gingiva, periodontal ligament, alveolar bone, and cementum. The periodontal ligament, which connects the alveolar bone to the root cementum and suspends the tooth in its alveolus, contains stem cells with the potential to form periodontal structures such as cementum and ligament [39]. The periodontal ligament contains fibroblasts, cementoblasts, osteoblasts, macrophages, undifferentiated ectomesenchymal cells, cell rests of Malassez, and vascular and neural elements that are capable of generating and maintaining periodontal tissues [40]. PDL-SCs express the MSC-associated markers CD13, CD29, CD44, CD59, CD90, and CD105, as well as STRO-1 [41]. Similar to other MSCs, PDL-SCs show osteogenic, adipogenic, and chondrogenic characteristics under defined culture conditions in vitro [42–44].

## 5. Trabecular Bone-Derived-Stem Cells (TB-MSCs)

The pioneering studies on human TB-MSCs were carried out by Beresford et al. [45], MacDonald et al. [46], Wergedal and Baylink [47], and Robey and Termine [48]. Tuli et al. isolated a CD73^+^, STRO-1^+^, CD105^+^, CD34^−^, CD45^−^, CD144^−^ cell population from human bone fragments. These cells exhibited stem cell-like characteristics such as a stable undifferentiated phenotype, and the ability to proliferate extensively and differentiate into osteoblastic, adipogenic and chondrogenic lineages [10, 49]. Thus, these cells were named human trabecular bone mesenchymal progenitor cells [10]. In another study, Sottile et al. demonstrated that cultures of TB-MSCs are equivalent to cultures of bone marrow-derived stem cells in terms of proliferation and multipotent differentiation capabilities [49]. Since the first description, in vitro secondary culture of cells derived from human trabecular bone have been used to examine implant-bone interactions and osteoblast biology [10, 49].

## 6. Synovial Membrane-Derived Stem Cells (SM-MSCs)

The synovial membrane is a source of relatively homogeneous, fibroblast-shaped, multipotent MSCs [9, 50]. The protocol used for isolating MSCs and fibroblasts from synovial membranes is the same [51]; however, SM-MSCs have a phenotype very similar to that of type B synoviocytes, that is, they contain characteristic lamellar bodies and express surfactant protein A, a hydrophilic protein also found in lung surfactant [50]. Fluorescent-activated cell sorting (FACS) analysis revealed that SM-MSCs are CD34^−^, CD45^−^, CD31^−^, CD14^−^ and CD44^+^, CD73^+^, CD90^+^, CD105^+^, a phenotype similar to that of MSCs derived from other tissues [9, 51, 52]. SM-MSCs are immunosuppressive and differentiate into chondrogenic, adipogenic, and, to a lesser extent, osteogenic and myogenic lineages [9, 51]. Yoshimura et al. found that rat SM-MSCs were superior to bone-marrow-, adipose tissue-, periosteum-, and muscle-derived stem cells in terms of colony number per nucleated cell, colony number per adherent cell, and cell number per colony [37]. In particular, SM-MSCs showed the highest potential for chondrogenic differentiation, making them an ideal MSC type for cartilage regeneration studies in rat models [37]. Similar findings were reported [52] for human MSCs derived from bone marrow, synovium, periosteum, skeletal muscle, and adipose tissue. Synovium can be harvested arthroscopically with a relatively low level of invasiveness. Donor site morbidity is also low due to the high regenerative capacity of the synovial membrane [52].

## 7. Periosteum-Derived Stem Cells (P-MSCs)

P-MSCs are essential for bone repair and a reduction in the availability of P-MSCs leads to a significant decrease in the healing capacity of bone [53]. Yoshimura et al. [37] found that rat P-MSCs showed the highest osteogenic differentiation potential. The osteogenic potential of P-MSCs is further supported by Perka et al. [54], who used P-MSCs seeded into polyglycolid-polylactid acid scaffolds to treat ulnar defects in New Zealand white rabbits. P-MSCs share a common surface marker expression profile with other MSCs; thus, they are CD11^−^, CD45^−^, and CD90^+^. Johnstone et al. [55] successfully repaired an experimental cartilage defect using P-MSCs, thereby demonstrating their capacity to differentiate into different cell lineages.

## 8. Muscle-Derived Stem Cells and Satellite Cells (M-MSCs)

Postnatal skeletal muscle tissue, similar to bone marrow, contains two different types of stem cells, M-MSCs and satellite cells, both of which can function as muscle precursors [56, 57]. Satellite cells are unipotent cells that originate from a population of muscle progenitors during embryogenesis [58]. The origin of satellite cells is among the most thoroughly studied aspects of morphogenesis. Segmental mesodermal structures on each side of the neural tube give rise to the skeletal muscle of the body [58]. A number of studies show that satellite cells from the trunk and extremities originate from the central and lateral dermomyotome, respectively, while those in the head originate from head mesoderm. Satellite cells in an adult constitute a small fraction of cells (2–7%) relative to the number of cells that fused to generate a particular muscle fiber [58], but are necessary for postnatal muscle regeneration [13, 56, 57]. A small subpopulation of satellite cells are stem cells by definition, since they possess an inherent capacity for self-renewal and can give rise to daughter cells [56, 57]. Satellite cells maintain a close spatial relationship with the muscles from which they derive, occupying the grooves or depressions between the basal lamina and sarcolemma, which suggests that a local source, rather than a distant one, produces the satellite cells [56, 58]. The hallmark genes for satellite cells are Pax 7 and Pax 3, with the latter only being expressed by a subset of satellite cells [58].

M-MSCs not only act as muscle precursors but also give rise to a variety of other cell types, including hematopoietic cells [56, 59, 60]. M-MSCs have a high proliferation and self-renewal capacity and are CD34^+^, Sca1^+^, CD45^−^, and c-Kit^−^ [57]. M-MSCs are capable of differentiating into skeletal muscle cells both in vivo and in vitro and spontaneously express myogenic markers. Taken together, these data suggest that M-MSCs are derived from skeletal myofibers [57]. However, a recent study by McKinney-Freeman et al. [61] suggests that M-MSCs are, in fact, HSCs residing in skeletal muscle rather than transdifferentiated myogenic cells.

## 9. Skin Stem Cells (SSCs)

MSCs are found in the dermal layer of skin. Toma et al. [14] isolated a multipotent, nestin and fibronectin positive, adult stem cell population from rodent skin. In a recent study by Vishnubalaji et al. mesenchymal stem cells isolated from human dermal skin were positive for CD105, CD90, CD73, CD29, CD13, and CD44 and were negative for endothelial and hematopoietic lineage markers CD45, CD34, CD31, CD14, and HLA DR [62]. Shi and Cheng [63] added that MSCs from newborn dermis were also positive for CD59, vascular cell adhesion molecule-1 (VCAM-1), and intercellular adhesion molecule-1 (ICAM-1). Other surface markers that are reported to be expressed by SSCs are CD49, CD166, SH2, SH4, EGFR, PDGFRa [64], CD271 [65], Stro-1 [66], CD71, CD133, and CD166 [67]. SSCs can differentiate into adipocytes, osteoblast, chondrocyte, neuron, hepatocyte, and insulin producing pancreatic cells [62].

## 10. Wharton's Jelly Stem Cells (WJ-MSCs)

WJ-MSCs are obtained from Wharton's jelly of umbilical cord [68]. Compared to BM-MSCs, WJ-MSCs exhibit a higher expression of undifferentiated human embryonic stem cell (hES) markers like NANOG, DNMT3B, and GABRB3 [69]; thus, they are more primitive then other types of MSCs and easy to obtain with no ethical considerations [70]. They express the typical MSCs markers: CD105, CD73, and CD90 and negative for CD45, CD34, CD14, CD19, and HLA-DR [70]. UC-MSCs can be induced into endothelial cells, adipogenic, osteogenic, chondrogenic, neurogenic lineages [70], insulin producing cells [71], and hepatocyte-like cells [72].

## 11. Miscellaneous Stem Cells

MSCs reside in essentially all adult tissues [73]. In addition to the MSCs discussed in this paper, stem cells have been isolated from liver, perichondrium, pancreas, hair follicles, intestinal epithelium, placenta, and amniotic membranes.

## 12. Clinical Use and Future Perspectives

Using stem cells alone, or in combination with scaffolds, to regenerate organs or tissues is a quite new idea. The type of cell and the route of administration are both equally important for the success of such stem cell treatments. ES cells show the greatest potential for tissue regeneration owing to their totipotency. Barberi et al. [74], Benninger et al. [75], and Chiba et al. [76] reported that ES cell-derived neurons injected into the mouse brain were successfully integrated and corrected the phenotype of a neurodegenerative disease. However, the clinical use of ES cells is encumbered by ethical considerations.

In a comparative study of human MSCs obtained from various tissues, Sakaguchi et al. [52] demonstrated that SM-MSCs and ASCs show superior adipogenic potential, whereas BM-MSCs, SM-MSCs, and P-MSCs show superior osteogenic potential. ASCs are a relatively new subtype of MSC that can be obtained by less invasive methods and in larger quantities than other MSCs [19]. ASCs also have a multilineage differentiation capacity similar to that of BM-MSCs and can easily be grown in standard tissue culture conditions [19]. The above data provide a useful guide for selecting the appropriate type of MSC for use in clinical regenerative medicine.

The growth factor secretome of hMSCs was characterized by Haynesworth et al. [36]. The secretory activity of hMSCs helps them to establish a regenerative microenvironment at sites of tissue injury [2]. Nevertheless, there is no evidence for a biologically significant effect of systemic MSC injection [77]. Tissue engineering offers a number of scaffolds to improve the outcomes of stem cell applications, enabling more precise targeting of transplanted MSCs. Scaffolds can be used in a number of different ways: (i) scaffolds can be seeded with MSCs in vitro and implanted after a short incubation period, (ii) loaded scaffolds can be kept in differentiation medium for 1–2 weeks before implantation to stimulate MSCs to differentiate into a specific lineage, or (iii) MSCs can be induced into a specific lineage before seeding into the scaffold and the scaffold implanted shortly thereafter [78]. The organization of the cells on the scaffolds and formation of vascular channels can be induced using specific growth factors, but this requires an adequate understanding of the paracrine mechanisms controlling tissue growth [16]. These complex paracrine mechanisms have attracted a great deal of interest in recent years, but are still not fully understood.

The tissues that can readily be engineered using stem cells are skin [79], cornea [80], bone [81], blood vessels [82], cartilage [83], dentin [84], heart muscle [85], liver [86], pancreas [87], nervous tissue [88], skeletal muscle [89], and tendon [90, 91]. Given the experimental data collected thus far, tissue engineered cardiac muscle [16], bone [81], and cartilage [92] seem to be the most suitable candidates for routine clinical application. BM-MSC transplantation improves cardiac function in patients with myocardial infarction and no side effects have been reported [93]. An alternative method for MSC transplantation is the cell sheet method [94]. Osiris Therapeutics in the USA launched a phase 1 safety trial of autologous hMSCs, which are delivered on a hydroxyapatite implant for alveolar ridge regeneration prior to dental implantation [92]. The same tissue engineering techniques have been applied to cartilage tissue engineering, enabling our group to construct ear cartilage in vitro [92]. MSCs have also been employed for cartilage regeneration in rheumatoid arthritis and osteoarthritis, but the results were not satisfactory [95]. Complete in vivo restoration of cartilage has not yet been achieved.

A search using NIH database (http://clinicaltrials.gov/) yielded 218 ongoing clinical trials utilizing MSCs. The main indications are multiple sclerosis, type I diabetes, GVHD, inflammatory bowel disease, cardiac ischemic diseases, cerebral vascular diseases, various autoimmune connective tissue disorders, spinal cord injury, ischemic extremity diseases, liver diseases and, bone and cartilage defects. A potential clinical use of MSCs is related to their anti-inflammatory and immunosuppressive effects. The results of phase III and phase II clinical trials conducted by Osiris Therapeutics and Le Blanc et al., respectively, suggested that BM-MSCs block acute graft versus host disease (GVHD) without any side effects [96, 97]. An additional clinical trial carried out by Osiris Therapeutics showed improvements in the symptoms of the inflammatory bowel disease (Crohn's disease) by at least 100 points on the Crohn's disease activity index [96]. A phase I study by Duijvestein et al. yielded similar results [98]. There a number of studies reporting the usefulness of MSCs in the treatment of autoimmune disorders such as rheumatic diseases, autoimmune encephalomyelitis, multiple sclerosis, and systemic lupus erythematosus (SLE) [99–103]. MSCs are believed to exhibit their immunomodulatory effects through soluble factors [104, 105]. In the light of the data obtained from experimental studies some clinical trials were launched and a consensus on the use of MSCs for multiple sclerosis has already been established [106, 107]. Another clinical use of MSCs is for the immune-modulation following solid organ transplants [108].

Once considered rather futuristic, in utero human HSC transplantation has become technically feasible and may be the gold standard for treating congenital hematological diseases and enzyme deficiencies [109].

Muscular dystrophies constitute a special group of disorders that may also be treated with MSCs. Muscular dystrophy defines a heterogeneous group of muscular disorders characterized by deficient production of dystrophin, which links the actin cytoskeleton to the extracellular matrix protein laminin, thereby protecting the muscle fibers from contraction induced damage [56]. Loss of dystrophin leads to damage to the sarcolemma that in turn leads to continued activation of satellite cells [56]. Repeated cycles of degeneration and regeneration overwhelm the regenerative capacity of the satellite cells, causing muscular weakness as the disease progresses [56]. Stem cell transplantation to restore the defective dystrophin is a promising treatment modality [56]. However, there are conflicting reports regarding the success of stem cell transplantation. In a study performed to investigate whether HSCs can participate in muscle regeneration, transplanted BM-HSCs were poorly engrafted in dystrophic muscles and restored dystrophin expression only in an average of 0.23% of fibers [110]. By contrast, Qu-Petersen et al. [57] reported successful M-MSC transplantation into mdx mice, even though the animals were not immunosuppressed. The number of M-MSCs found in the mdx muscle was stable over a 90-day period. Interestingly, the results could not be reproduced using satellite cells. This was attributed to the lack of expression of MHC-I by M-MSCs, thereby granting them immune-privilege, and to the higher self-renewal ability of M-MSCs. To date, clinically useful levels of stem cell engraftment to muscle tissue have not been reported [56]. Alternatively, stem cells could be used to introduce genes into muscle tissue to increase the production of deficient proteins [5, 56]. MSCs can easily be obtained from patients, manipulated genetically, expanded to obtain an adequate number of cells and, finally, reintroduced into body [5]. This treatment schema bypasses the risks associated with virus vectors [5]. However, the current level of gene transduction into MSCs, the level of engraftment of MSCs to the target tissues, and the sustainability of the desired gene expression are the main issues that need to be improved if this is to become an effective treatment modality [5]. Furthermore, genetically modified MSCs may not necessarily incorporate into target tissues to correct the defective gene, but may also reside in the connective tissue acting as minipumps that secrete the gene product [5]. Methods to increase the efficiency of gene therapy are currently ongoing.

The current problems with the clinical application of MSCs are insufficient engraftment of the stem cells to target tissues, inadequate vascularization of tissue engineered constructs to ensure long term viability, the possibility of inducing teratomas [16], and immunogenic reactions directed against allogeneic cells [16]. In addition, the expression of one or more proteins specific for a certain cell lineage in vitro does not necessarily mean that MSCs bearing these proteins will exhibit the functions of this specific cell type properly in vivo [111]. Moreover, the study by Terada et al. raises doubts regarding whether in vivo transdifferentiation of transplanted MSCs actually occurs, or is the result of cell fusion misinterpreted as transdifferentiation [92, 112]. Safety issues regarding the MSCs transplantation have been largely solved, particularly with autologous transplants; however, sustained curative benefit has not been established yet. Increasingly, new stem cell types are being explored and there are a considerable number of clinical phase I/II trials as mentioned previously. Even though it is too early to predict the outcome of these trials at present, early observations of patients indicate promising results without any significant side effects [113]. Transfer of xenogenic proteins into human body along with the MSCs is another potential problem in clinical use of MSCs. Main source of xenogenic contamination is the fetal bovine serum (FBS) used as a supplement for in vitro expansion of MSCs. FBS should be replaced with an autologous or xeno-free supplement in the clinical setting [114, 115]. As new information is gathered from future studies, our understanding of the complex differentiation mechanisms of stem cells will help us to solve current problems and achieve crucial improvements in the use of stem cells for clinical applications.

## 13. Conclusion

There is no doubt that stem cell therapy is a promising treatment for the regeneration of damaged human tissues. Some successful clinical results have been reported by a number of groups. However, the methods of administration need to be improved before a broader spectrum of clinical applications can be successfully achieved. Currently, the possibility of obtaining a significant clinical outcome after systemic administration of MSCs without specific targeting seems remote.

## Figures and Tables

**Table 1:** Surface marker expression profiles of main MSCs types.

MSCs	CD marker expression*
ASCs	CD13^+^, CD29^+^, CD44^+^, CD71^+^, CD90^+^, CD105/SH2 and SH3^+^, STRO-1^+^.
BM-MSCs	CD44^+^, CD105^+^, CD166^+^, CD28^+^, CD33^+^, CD13^+^, HLA class I^+^
ES	SSEA 3&4^+^, CD90^+^, CD9^+^, TRA-1-60^+^, TRA-1-81^+^, GCTM2^+^, GCT343^+^, TRA-2-54^+^, TRA-2-49^+^, class I HLA^+^
HSCs	CD34^+^, CD90^+^
PDLSCs	STRO-1^+^, CD13^+^, CD29^+^, CD44^+^, CD59^+^, CD90^+^, CD105^+^
TB-MSCs	CD73^+^, STRO-1^+^, CD105^+^
SM-MSCs	CD44^+^, CD73^+^, CD90^+^, CD105^+^
Periosteum-MSCs	CD90^+^
M-MSCs	CD34^+^, Sca1^+^
Dermal SSCs**	CD105^+^, CD90^+^, CD73^+^, CD29^+^, CD13^+^, CD44^+^CD59^+^, VCAM-1^+^, ICAM-1^+^, CD49^+^, CD166^+^, SH2^+^, SH4^+^, EGFR^+^, PDGFRa^+^, CD271^+^, Stro-1^+^, CD71^+^, CD133^+^, CD166^+^
WJ-MSCs	CD105^+^, CD73^+^, CD90^+^

*MSCs are commonly negative for CD14, CD16, CD31, CD34, CD45, CD 56, CD61, CD62E, CD104, and CD106.

**There is still no consensus regarding the location, markers, and subgroups of human epidermal skin stem cells.
